# Non-small Cell Lung Cancer Cells Modulate the Development of Human CD1c^+^ Conventional Dendritic Cell Subsets Mediated by CD103 and CD205

**DOI:** 10.3389/fimmu.2019.02829

**Published:** 2019-12-10

**Authors:** Yong Lu, Wenlong Xu, Yanli Gu, Xu Chang, Guojian Wei, Zhien Rong, Li Qin, Xiaoping Chen, Fang Zhou

**Affiliations:** ^1^State Key Laboratory of Respiratory Disease, National Clinical Research Center for Respiratory Disease, Guangzhou Institute of Respiratory Health, The First Affiliated Hospital of Guangzhou Medical University, Guangzhou, China; ^2^Department of Experimental and Clinical Immunology, CAS Lamvac Biotech Co., Ltd., Guangzhou, China; ^3^Center of Infection and Immunity, Guangzhou Institutes of Biomedicine and Health, Chinese Academy of Sciences, Guangzhou, China

**Keywords:** dendritic cell, immune tolerance, immunotherapy, non-small lung cancer, CD1c^+^ cDCs

## Abstract

Advanced non-small cell lung cancer (NSCLC) leads to a high death rate in patients and is a major threat to human health. NSCLC induces an immune suppressive microenvironment and escapes from immune surveillance *in vivo*. At present, the molecular mechanisms of NSCLC immunopathogenesis and the immune suppressive microenvironment induced by NSCLC have not been fully elucidated. Here, we focus on the effect of NSCLC cells on the development and differentiation of human CD1c^+^ conventional dendritic cell (DC) subsets mediated by CD205 and CD103. The peripheral blood mononuclear cells (PBMCs) were isolated from NSCLC patients and healthy donors. DCs were induced and cocultured with primary NSCLC cells or tumor cell line H1299. DCs without incubation with tumor cells are control. The protein expression of costimulatory molecules such as CD80 and CD86, HLA-DR, pro-/anti-inflammatory cytokines such as IL-10 and IL-12, and CD205 and CD103 on CD1c^+^ DCs was detected by flow cytometry. Our data revealed two new subpopulations of human CD1c^+^ DCs (CD1c^+^CD205^+^CD103^+^ and CD1c^+^CD205^+^CD103^−^ DC) in healthy donors and NSCLC patients. NSCLC cells modulate the development of the CD1c^+^CD205^+^CD103^+^ DC and CD1c^+^CD205^+^CD103^−^ DC subpopulations *in vitro* and *ex vivo*. NSCLC cells also suppress the expression of signal molecules such as CD40, CD80, CD86, and HLA-DR on CD1c^+^ DCs. In addition, the production of pro-inflammatory cytokines, including IL-12 and IL-23, is downregulated by NSCLC cells; however, the secretion of anti-inflammatory cytokines, such as IL-10 and IL-27, by CD1c^+^ DCs is upregulated by NSCLC cells. Our results suggest that NSCLC cells may induce immune tolerogenic DCs, which block DC-mediated anti-tumor immunity in NSCLC patients. Our data may be helpful in revealing new cellular mechanisms related to the induction of tolerogenic CD1c^+^ DCs by NSCLCs and the development of an immune suppressive microenvironment that causes tumor cells to escape immune surveillance. Our results indicate a potential role for CD1c^+^ DC subsets mediated by CD205 and CD103 in DC-mediated immunotherapy to target NSCLC in the future.

## Introduction

Non-small cell lung cancer (NSCLC) is a major type of lung cancer (1–3). The survival rate of late-stage NSCLC is very low (4). At present, the immunopathogenesis of NSCLC has not been fully elucidated (5). NSCLC cells escape from immune surveillance *in vivo* and induce a tumor immune suppressive microenvironment (6). The molecular mechanisms involved in the NSCLC-induced tumor immune suppressive microenvironment are still unknown (7). We focused on the effect of NSCLC cells on dendritic cell (DC)-mediated immune function in this research project. We propose that NSCLC cells may induce specific immune tolerogenic DCs and suppress DC-mediated immune responses *in vivo*. Our results will show that NSCLC cells inhibit the expression of signal molecules such as CD40, CD80, and CD86 on DCs. In addition, NSCLC cells also regulate the production of multiple pro- and anti-inflammatory cytokines, such as IL-6, IL-10, IL-12, IL-23, IL-27, and TGF-β, in DCs. NSCLC cells may affect the immune function of DCs mediated by these signal molecules and cytokines *in vivo*.

DCs are major regulatory immune cells that are necessary for adaptive and innate immunity (8, 9). DCs comprise at least two typical types: conventional DCs (cDCs) and plasmacytoid DCs (pDCs) (10, 11). In addition, DCs can also be divided into inflammatory and tolerogenic DCs according to their different immune functions (12, 13). There are at least three subsets of DCs in human peripheral blood mononuclear cells (PBMCs): CD1c^+^ (cDCs), CD141^+^ (cDCs), and CD303^+^ DCs (pDCs) (14). Their immune functions have not yet been fully elucidated. In this project, the effect of NSCLC cells on the expression of signal molecules and cytokine production in CD1c^+^ DCs was investigated. Our results suggest that NSCLC cells may induce immune tolerogenic DCs through modulating the expression and production of signal molecules and cytokines in CD1c^+^ DCs, which play an important role in anti-tumor immunity and immune tolerance *in vivo*.

CD1c^+^ DCs are cDCs in human peripheral blood (15). At present, the functions of the CD1c^+^ DC subsets in humans have not been fully elucidated (16). It is still unknown whether NSCLC cells can modulate the development and differentiation of CD1c^+^ DC subsets, although Stankovic et al. investigated DC composition in NSCLC patients (17). Tabarkiewicz et al. reported that the percentage of CD1c^+^ DCs in NSCLC patients is lower than that in healthy donors (18). It is unclear whether NSCLC cells affect the development and differentiation of CD1c^+^ DC subpopulations. In this study, two new subsets of CD1c^+^ DCs with activity mediated by CD205 and CD103 were found in both healthy donors and NSCLC patients. NSCLC cells modulate the development and differentiation of CD1c^+^ DC subpopulations, and this is mediated by CD205 and CD103. Our results imply that NSCLC cells may affect the immune function of CD1c^+^ DC subsets via regulating the expression of CD205 and CD103 on CD1c^+^ DCs. This is likely one aspect of the cellular mechanisms involved in the NSCLC-induced immune suppressive microenvironment *in vivo*.

## Materials and Methods

### Patients and Healthy Donors

All patients and healthy donors were recruited via the CAS Lamvac Biotech Co., Ltd. registry and provided informed consent. PBMCs were obtained from seven patients and seven healthy donors. The cells have been collected and studied since 2017. The details of the characteristics of the NSCLC patients and healthy donors are summarized in Supplementary Table 1. All samples were tested in the CAS Lamvac Biotech Co., Ltd. Animal and Human Care facilities, and all experimental procedures were approved by the Institutional Animal and Human Care and Use Committee of Cas Lamvac Biotech Co., Ltd.

### Isolation of Human PBMCs

Human blood samples (5 ml blood obtained from each person) were centrifuged at 300 g for 20 min at room temperature (RT). The plasma was transferred into a clean, labeled 15-ml conical tube for each sample with a 5-ml pipet after centrifugation. The buffy coat, including lymphocytes, was then transferred into a new clean 15-ml conical tube with a 2-ml pipet using a circular motion. The buffy coat was diluted 1:3 with 1× sterilized PBS and inverted at RT. The diluted buffy coat was then slowly and carefully transferred into 3 ml of Lympholyte-H (Cedarlane Laboratories Limited, Burlington, ON, Canada) with a 10-ml pipet at RT. The cells were then centrifuged at 800 *g* for 20 min at RT. The cells in the lymphocyte layer were transferred into a new 50-ml conical tube by using a 2-ml serological pipet. The lymphocytes were then diluted with 40 ml staining buffer (5% fetal bovine serum, FCS, Gibco, Grand Island, NY, USA, and 0.1% azide in 1 × sterilized PBS). The cells were then centrifuged twice at 500 *g* for 10 min at RT. The supernatant was decanted. The PBMCs were then diluted with 5 ml of media A (40% heated inactive human AB serum in RPMI 1640 medium, Sigma, St. Louis, MO, USA) for the FACS assay.

### Freezing and Thawing of PBMCs

The total PBMCs were counted, and 3 × 10^6^ cells were placed into each cryo-vial tube along with 0.5 ml of media A. Then, 0.5 ml of media B (20% DMSO in RPMI 1640 medium, Sigma) was added to each cryo-vial tube. The cryo-vial tubes were then sealed and placed into a cell freezing container containing isopropanol. The cells were kept at −80°C for 24 h and then put into a liquid nitrogen (LN2) canister with LN2.

When thawing frozen PBMCs, the frozen cells were quickly thawed at 37°C for 1 min. Cells were resuspended in RPMI 1640 complete medium with benzonase (25 U/ml) (Sigma). The PBMCs were then centrifuged twice at 300 *g* for 8 min. Finally, the cells were resuspended in 1 ml of complete RPMI 1640 medium (Gibco) without benzonase for counting, and the cell concentration was adjusted with complete RPMI 1640 medium without benzonase for the flow cytometry assay.

### Human DC Culture

A total of 1 × 10^7^ PBMCs in 5 ml of RPMI 1640 complete medium were placed into T_25_ flasks and incubated at 37°C with 5% CO_2_ for 4 h. The floating cells were removed, and the attached mononuclear cells were incubated with DC culture medium (complete medium with 1,000 IU/ml GM-CSF and 500 IU/ml IL-4, PeproTech, Rocky Hill, NJ, USA) at day 0. Half of the DC culture medium was removed on days 3 and 6. The DCs were then centrifuged twice at 300 g for 5 min. The supernatant was decanted, and the cells were resuspended in the same amount of fresh DC culture medium and placed into the same DC culture flask. The DCs were harvested at day 8 for the flow cytometry assay.

### Tumor Cell Line and Primary NSCLC Cell Culture

Tumor tissues and para-carcinoma tissues were resected and sterilized. The histologically malignant tissue and para-cancerous tissue were washed with PBS three times. The tissues were cut and ground using a sterilized sieve (*d* = 0.075 mm). The primary human tumor cells and human H-1299 non-small lung cancer cells (Cell Bank, Chinese Academy of Sciences, P.R. China) were resuspended in RPMI 1640 complete medium for the flow cytometry assay.

### Flow Cytometry Assay

For surface staining, 5 × 10^5^ DCs were either incubated with living tumor cells or were not cocultured with tumor cells, and all cells were stained with BV 480-human CD40 (Becton Dickinson, BD; Franklin Lakes, NJ, USA), BV 650-human CD80 (Biolegend, San Diego, CA, USA), BV 605-human CD86 (BD), APC-Cy7-human CD1c (Biolegend), BV 711-human CD103 (Biolegend), BV 421-human CD205 (BD), AF 700-human HLA-DR (eBiosciences, Grand Island, NY, USA), and BV 510 lineage antibodies (Lin) (Biolegend) for 24 h at 4°C. The cells were washed twice with staining buffer (Biolegend) at 300 g for 5 min. The DCs were fixed with 0.3 ml of fixation buffer (Biolegend) per sample for 15 min in a dark room at RT. The cells were then centrifuged twice with a permeabilization buffer (Biolegend) at 800 g for 10 min. Finally, the cells were resuspended in 0.1 ml of permeabilization buffer per sample for intracellular staining.

For intracellular staining, DCs were incubated with FITC-human IL-6 (Biolegend), Pacific Blue-human IL-12 (Biolegend), BV 786-human IL-10 (BD), PE-CF594-human TGF-beta1 (BD), PE-human IL-27 (Biolegend), and eFluor 660-human IL-23p19 antibodies (eBiosciences) for 24 h at 4°C. The cells were centrifuged twice with permeabilization buffer at 800 *g* for 5 min and resuspended in 0.3 ml of staining buffer per sample. The cells were analyzed by a Cytek Aurora flow cytometry instrument (Cytek Biosciences Inc., Fremont, CA, USA). The flow cytometry assay data were analyzed using Flow Jo software (TreeStar, Ashland, OR, USA).

### Statistical Analysis

Experimental data were analyzed by Prism software 6.0 (GraphPad Software, San Diego, CA, USA), and *t-*tests were conducted. The results were regarded as indicating a significant difference if the *P*-value was <0.05.

## Results

1. The development of the CD1c^+^CD103^+^CD205^+^ DC subset is suppressed in NSCLC patients.

**Figure 1:**
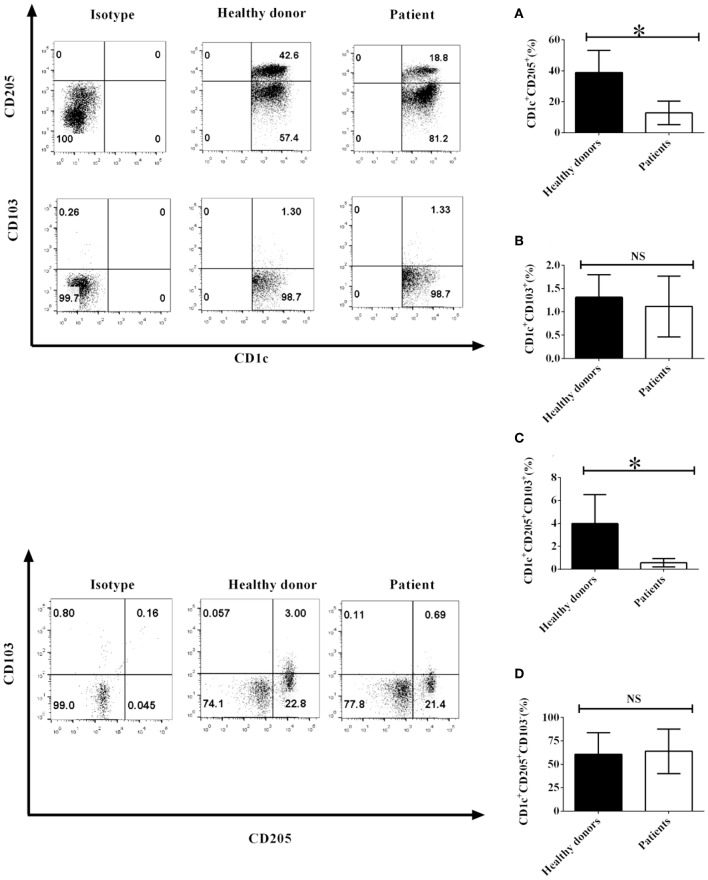
The phenotypes of CD1c^+^ DC subsets mediated by CD103 and CD205 in NSCLC patients and healthy donors. PBMCs from NSCLC patients and healthy donors were collected and stained with human CD1c, CD103, CD205, and lineage (Lin) antibodies. Lin^−^CD1c^+^ cells were gated like those shown in Supplementary Figure 1. Protein expression of CD205 **(A)** and CD103 **(B)** on CD1c^+^ DCs and the frequencies of CD1c^+^CD205^+^CD103^+^ DCs **(C)** and CD1c^+^CD205^+^CD103^−^ DCs **(D)** were determined. The error bars shown in this figure represent the mean and SD of quadruplicate determinations from one experiment (*P < 0.05, *n* = 4, *t*-test).

Since CD103 and CD205 expression on DCs play an important role in DC-mediated immune function, NSCLC cells may affect the biological function of DCs through modulating the expression of CD103 and CD205 on DCs. To investigate whether NSCLC cells regulate the expression of CD103 and CD205 on CD1c^+^ DCs, PBMCs were isolated from NSCLC patients and healthy donors. The expression of CD103 and CD205 on CD1c^+^ DCs was detected by flow cytometry. Our experimental results demonstrated that the number of CD1c^+^CD205^+^ DCs obtained from NSCLC patients was less than the number of CD1c^+^CD205^+^ DCs isolated from healthy donors (Figure 1A). In contrast, the number of CD1c^+^CD103^+^ DCs in NSCLC patients was similar to the number of CD1c^+^CD103^+^ DCs in healthy donors (Figure 1B). In addition, the population of the CD1c^+^CD103^+^CD205^+^ DC subset in NSCLC patients was also less than the population of the CD1c^+^CD103^+^CD205^+^ DC subset in healthy donors (Figure 1C). In contrast, there was no significant difference between healthy donors and NSCLC patients in the CD1c^+^CD205^+^CD103^−^ DC subpopulation (Figure 1D). This implies that NSCLC cells may modulate the development of the CD1c^+^ DC subset mediated by CD205 and CD103 *in vivo*.

2. H-1299 tumor cells regulate the development of CD1c^+^ DC subsets derived from NSCLC patients mediated by CD205 and CD103.

**Figure 2:**
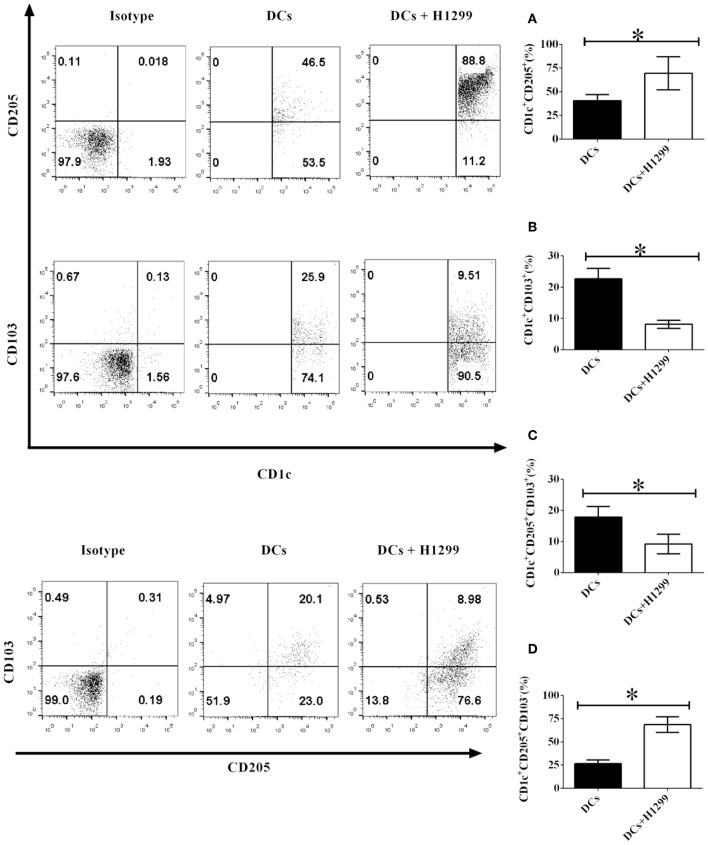
H-1299 tumor cells regulate the development of CD1c^+^ DC subpopulations derived from NSCLC patients mediated by CD205 and CD103. PBMCs from three NSCLC patients were collected and stained with human CD1c, CD103, CD205, and lineage antibodies. Lin^−^CD1c^+^ cells were gated. Protein expression of CD205 **(A)** and CD103 **(B)** on CD1c^+^ DCs was tested by flow cytometry. The frequencies of the CD1c^+^CD205^+^CD103^+^ DCs **(C)** and CD1c^+^CD205^+^CD103^−^ DCs **(D)** were determined. The error bars shown in this figure represent the mean and SD of triplicate determinations of the frequency of CD1c^+^ subpopulations in three independent experiments (*P < 0.05, *n* = 3, *t*-test).

Our data indicated that co-culture with H-1299 tumor cells modulates the development of CD1c^+^ DC subpopulations, which is mediated by CD205 and CD103, derived from healthy donors (Supplementary Figure 6). We proposed that H-1299 tumor cells may also regulate the differentiation of CD1c^+^ DC subsets isolated from NSCLC patients. To investigate this hypothesis, DCs isolated from three NSCLC patients were incubated with H-1299 tumor cells or were incubated without tumor cells as a control. The protein expression of CD205 (Figure 2A) and CD103 (Figure 2B) on CD1c^+^ DCs was detected by flow cytometry. Our data show that coculture with H-1299 cells upregulated the expression of CD205 but downregulated the expression of CD103 on CD1c^+^ DCs compared with that of those on CD1c^+^ DCs that were not cocultured with H-1299 tumor cells (Figures 2A,B). In addition, incubation with H-1299 tumor cells suppressed the development of the CD1c^+^CD205^+^CD103^+^ DC subset, but it facilitated the differentiation of the CD1c^+^CD205^+^CD103^−^ DC subpopulation when compared with that of DCs that were not cocultured with H-1299 cells (Figures 2C,D). It can be concluded that coculture with H-1299 tumor cells modulates the development of CD1c^+^ DC subsets derived from NSCLC patients mediated by CD205 and CD103.

3. Primary NSCLC cells modulate the development and differentiation of CD1c^+^ DC subsets derived from NSCLC patients mediated by CD205 and CD103.

**Figure 3:**
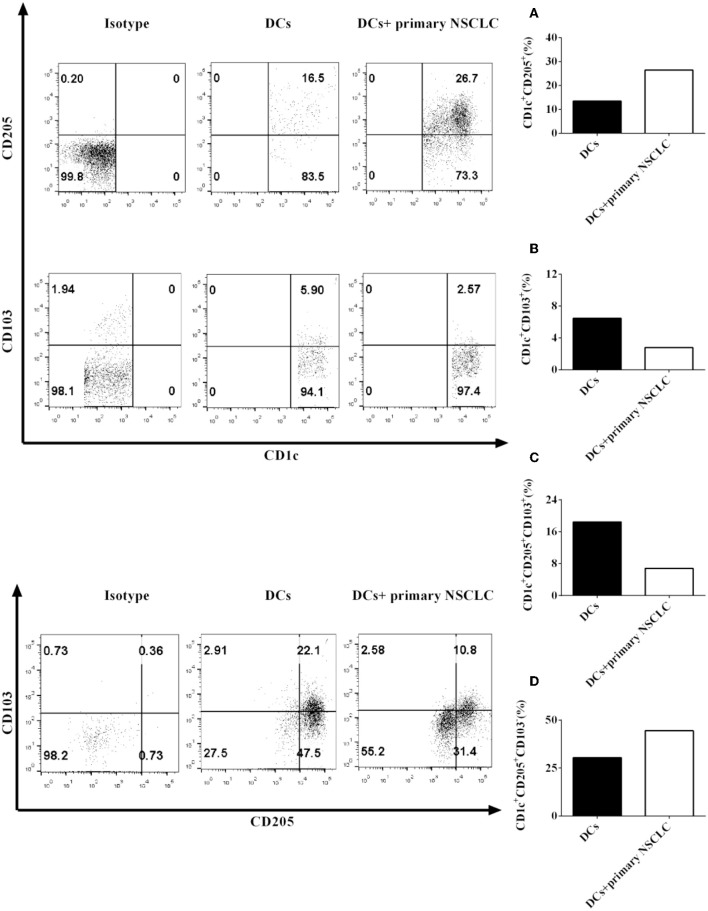
Primary NSCLC cells regulate the development of CD1c^+^ DC subpopulations derived from NSCLC patients mediated by CD205 and CD103. Primary tumor cells and PBMCs from two NSCLC patients were collected and stained with human CD1c, CD103, CD205, and lineage antibodies. Lin^−^CD1c^+^ cells were gated. Protein expression of CD205 **(A)** and CD103 **(B)** on CD1c^+^ DCs was detected by flow cytometry. The frequencies of the CD1c^+^CD205^+^CD103^+^ DC subset **(C)** and the CD1c^+^CD205^+^CD103^−^ DC subpopulations **(D)** were determined. The statistical figure shows the mean of determinations of the frequency of the CD1c^+^ subpopulations in two independent experiments (*n* = 2).

Our data showed that the NSCLC cell line H-1299 can modulate CD1c^+^ DC subset development mediated by CD205 and CD103 when they are cocultured with DCs derived from NSCLC patients (Figure 2). We hypothesized that primary NSCLC cells may also regulate the development of CD1c^+^ DC subpopulations through modulating the expression of CD205 and CD103 on DCs. To investigate this hypothesis, primary NSCLC cells were isolated from the cancer tissue from two NSCLC patients and cocultured with DCs derived from the same patients. The protein expression of CD205 (Figure 3A) and CD103 (Figure 3B) on CD1c^+^ DCs treated with primary tumor cells or without incubation with primary NSCLC cells was detected by flow cytometry. The experimental data indicate that the expression of CD205 on CD1c^+^ DCs was increased after coculture with primary tumor cells compared with that on CD1c^+^ DCs without incubation with primary NSCLC cells (Figure 3A). In contrast, CD103 expression on CD1c^+^ DCs incubated with primary NSCLC cells was downregulated compared with that on CD1c^+^ DCs without coculture with primary tumor cells (Figure 3B). In addition, coculture with primary NSCLC cells downregulated the differentiation of the CD1c^+^CD205^+^CD103^+^ DC subset compared with that of DCs without incubation with primary tumor cells (Figure 3C); however, incubation with primary tumor cells facilitates the development of the CD1c^+^CD205^+^CD103^−^ DC subpopulation compared with that without coculture with primary NSCLC cells (Figure 3D). It can be concluded that primary NSCLC cells also modulate the development and differentiation of CD1c^+^ DC subsets derived from NSCLC patients mediated by CD205 and CD103.

4. H-1299 tumor cells suppress the expression of signal molecules on CD1c^+^ DCs derived from NSCLC patients.

**Figure 4:**
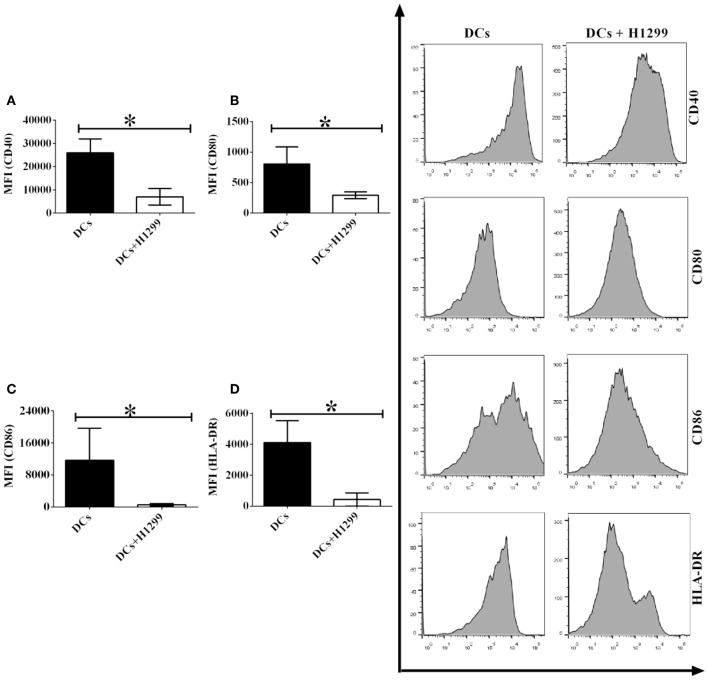
H-1299 tumor cells suppress the expression of signal molecules on CD1c^+^ DCs derived from NSCLC patients. PBMCs were isolated from three NSCLC patients and induced the development of DCs *in vitro*. DCs were incubated with H-1299 tumor cells for 24 h or were not cocultured with H-1299 cells as a control. DCs were stained with human CD1c, CD40, CD80, CD86, HLA-DR, and lineage antibodies. A flow cytometry assay was conducted, and Lin^−^CD1c^+^ cells were gated. Protein expression of CD40 **(A)**, CD80 **(B)**, CD86 **(C)**, and HLA-DR **(D)** on CD1c^+^ DCs is shown. The error bars indicated in this figure represent the mean and SD of triplicate determinations of the mean fluorescence identities (MFI) in three independent experiments (*P < 0.05, *n* = 3, *t*-test).

Since our results indicate that H-1299 tumor cells downregulate the expression of CD40, CD80, CD86, and HLA-DR on CD1c^+^ DCs isolated from healthy donors (Supplementary Figure 4), we proposed that H-1299 cells may also block the expression of costimulatory molecules on CD1c^+^ DCs derived from NSCLC patients. To investigate this hypothesis, DCs isolated from three NSCLC patients were incubated with H-1299 tumor cells or were not cocultured with H-1299 cells as a control. The protein expression of CD40 (Figure 4A), CD80 (Figure 4B), CD86 (Figure 4C), and HLA-DR (Figure 4D) was detected by flow cytometry. Our results demonstrated that the expression of CD40, CD80, CD86, and HLA-DR was downregulated after coculture with H-1299 tumor cells compared with that on CD1c^+^ DCs that were not incubated with H-1299 cells (Figure 4). It can be concluded that H-1299 tumor cells also suppress the expression of signal molecules on CD1c^+^ DCs derived from NSCLC patients, similar to their effect on CD1c^+^ DCs isolated from healthy donors (Supplementary Figure 4).

5. Primary NSCLC cells also inhibit the protein expression of signal molecules on CD1c^+^ DCs derived from NSCLC patients.

**Figure 5:**
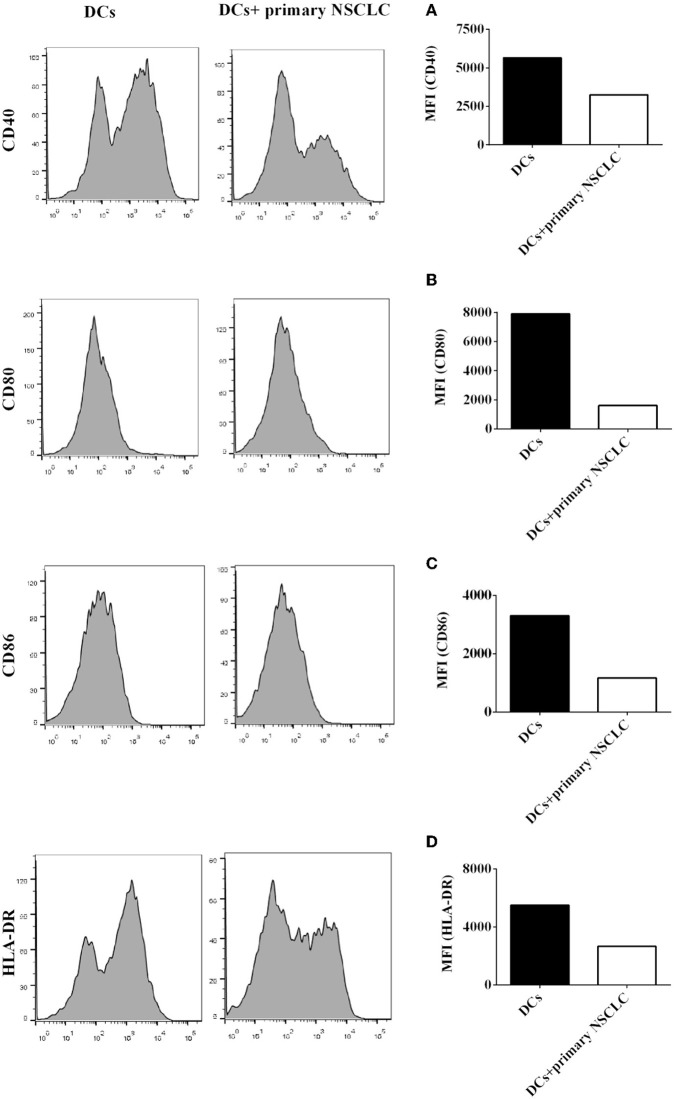
Primary NSCLC cells suppress the expression of signal molecules on CD1c^+^ DCs derived from NSCLC patients. Primary tumor cells were separated from tumor tissue of two NSCLC patients. PBMCs were also isolated from the same patients and induced the development of DCs *in vitro*. DCs were incubated with primary NSCLC cells for 24 h or were not cultured with primary tumor cells as a control. DCs were stained with human CD1c, CD40, CD80, CD86, HLA-DR, and lineage antibodies. A flow cytometry assay was conducted, and Lin^−^CD1c^+^ cells were gated. Protein expression of CD40 **(A)**, CD80 **(B)**, CD86 **(C)**, and HLA-DR **(D)** on CD1c^+^ DCs is shown. The statistical figure shows the mean of duplicate determinations of the mean fluorescence identities in two independent experiments (*n* = 2).

Our results demonstrated that coculture with H-1299 NSCLC cells leads to the downregulation of the expression of signal molecules, such as CD40, CD80, CD86, and HLA-DR, on CD1c^+^ DCs (Figure 4); however, H-1299 is a tumor cell line, and we are not certain whether primary NSCLC cells also suppress the expression of costimulatory molecules on DCs. To investigate whether primary NSCLC cells modulate the expression of signal molecules on CD1c^+^ DCs, primary tumor cells were isolated from tumor tissues of two NSCLC patients, and the primary tumor cells were incubated with DCs induced with PBMCs derived from the same patients. DCs without coculture with primary tumor cells served as a control. The protein expression of CD40 (Figure 5A), CD80 (Figure 5B), CD86 (Figure 5C), and HLA-DR (Figure 5D) on CD1c^+^ DCs was detected by flow cytometry. The experimental data showed that the protein expression of CD40, CD80, CD86, and HLA-DR on CD1c^+^ DCs was downregulated after co-culture with primary NSCLC cells compared with that on CD1c^+^ DCs that were not cocultured with tumor cells (Figures 5A–D). It can be concluded that primary NSCLC cells are able to downregulate the expression of CD40, CD80, CD86, and HLA-DR on CD1c^+^ DCs after incubation with DCs derived from the same NSCLC patients.

6. H-1299 tumor cells modulate the production of pro- and anti-inflammatory cytokines in CD1c^+^ DCs isolated from NSCLC patients.

**Figure 6:**
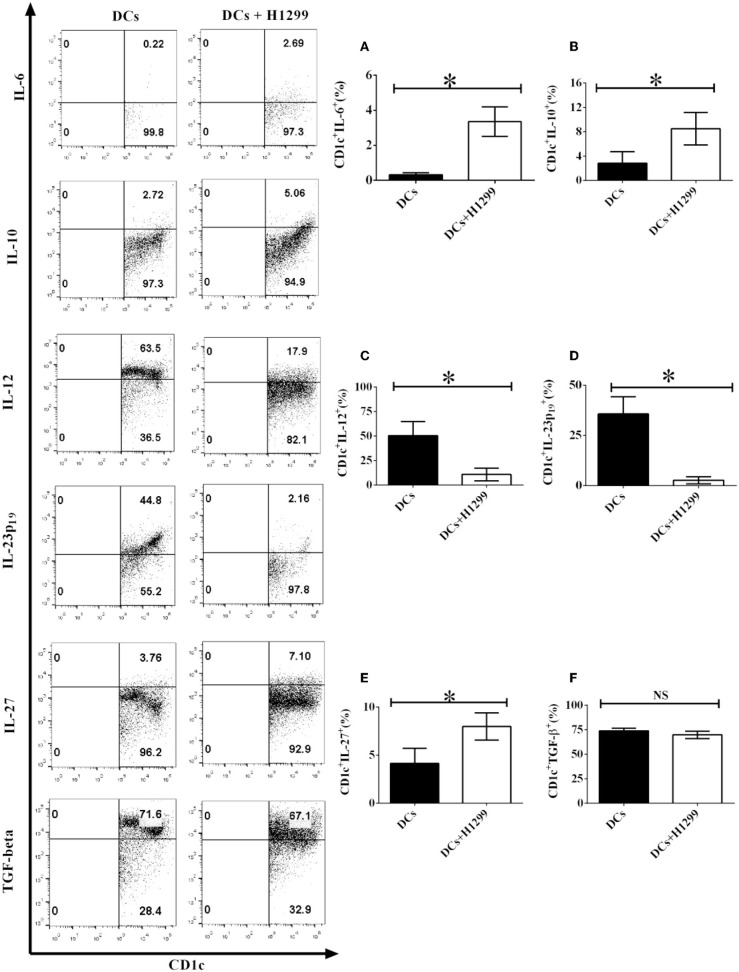
H-1299 tumor cells modulate the production of pro- and anti-inflammatory cytokines by CD1c^+^ DCs derived from NSCLC patients. PBMCs were obtained from three NSCLC patients. PBMCs were cultured in DC medium for 8 days to induce the development of DCs. DCs were stained with human CD1c, lineage, IL-6, IL-10, IL-12, IL-23 (p19), IL-27, and TGF-β antibodies. A flow cytometry assay was carried out and lin^−^CD1c^+^ cells were gated. Cytokine production, including IL-6 **(A)**, IL-10 **(B)**, IL-12 **(C)**, IL-23p19 **(D)**, IL-27 **(E)**, and TGF-β **(F)**, by CD1c^+^ DCs was determined. The error bars shown in this figure represent the mean and SD of triplicate determinations of the frequency of cytokine production by CD1c^+^ DCs in three independent experiments (*P < 0.05, *n* = 3, *t*-test).

Our data showed that H-1299 cells regulate the secretion of pro- and anti-inflammatory cytokines in CD1c^+^ DCs derived from healthy donors compared with those that were not cocultured with H-1299 cells (Supplementary Figure 5). We hypothesized that H-1299 cells may also affect the production of pro- and anti-inflammatory cytokines in CD1c^+^ DCs isolated from NSCLC patients. To test this hypothesis, DCs derived from three NSCLC patients were incubated with H-1299 tumor cells. DCs without incubation with H-1299 cells served as a control. Our results demonstrate that coculture with H-1299 tumor cells leads to the upregulation of IL-6, IL-10, and IL-27 production by CD1c^+^ DCs compared with that by CD1c^+^ DCs that were not cocultured with H-1299 cells (Figures 6A,B,E). In contrast, incubation with H-1299 cells causes the downregulation of IL-12 and IL-23 production by CD1c^+^ DCs compared with that by CD1c^+^ DCs that were not cocultured with H-1299 cells (Figures 6C,D). Moreover, H-1299 cells do not affect the production of TGF-β in CD1c^+^ DCs compared with that in CD1c^+^ DCs that were not cocultured with H-1299 cells (Figure 6F). These results are the same as those obtained with CD1c^+^ DCs derived from healthy donors, as shown in Supplementary Figure 5. It can be concluded that H-1299 tumor cells can modulate the production of pro- and anti-inflammatory cytokines by CD1c^+^ DCs derived from both healthy donors and NSCLC patients.

7. Primary NSCLC cells modulate the production of pro- and anti-inflammatory cytokines by CD1c^+^ DCs derived from NSCLC patients.

**Figure 7:**
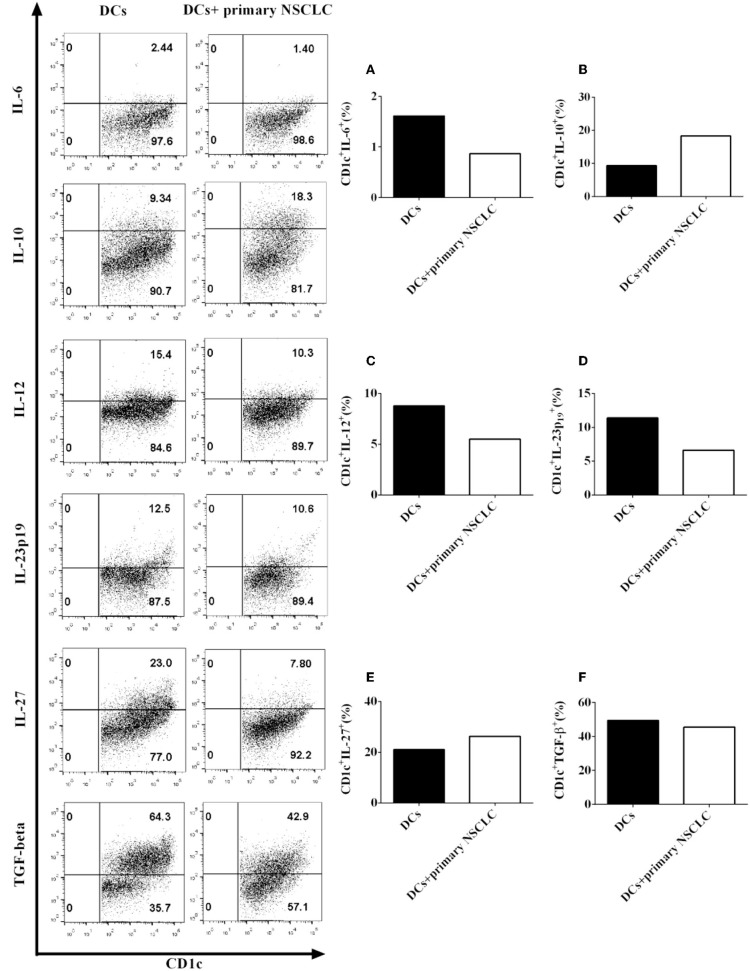
Primary NSCLC cells modulate the production of pro- and anti-inflammatory cytokines by CD1c^+^ DCs derived from two NSCLC patients. Primary tumor cells and PBMCs were isolated from the same NSCLC patients. PBMCs were cultured in DC medium for 8 days to induce the development of DCs. DCs were stained with human CD1c, lineage, IL-6, IL-10, IL-12, IL-23 (p19), IL-27, and TGF-β antibodies. A flow cytometry assay was carried out, and lin^−^CD1c^+^ cells were gated. The cytokine production of IL-6 **(A)**, IL-10 **(B)**, IL-12 **(C)**, IL-23 (p19) **(D)**, IL-27 **(E)**, and TGF-β **(F)** by CD1c^+^ DCs was determined. The statistical figure shows the mean of duplicate determinations of cytokine production by CD1c^+^ DCs in two independent experiments (*n* = 2).

Since our data show that the NSCLC cell line H-1299 regulates the secretion of multiple cytokines by CD1c^+^ DCs (Figure 6), we propose that primary NSCLC cells may also affect the production of pro- and anti-inflammatory cytokines by DCs *in vivo*. To test this hypothesis, primary NSCLC cells were isolated from tumor tissue and cocultured with DCs derived from the same NSCLC patients. DCs that are not cocultured with primary tumor cells served as a control. The production of the cytokines IL-6 (Figure 7A), IL-10 (Figure 7B), IL-12 (Figure 7C), IL-23 (Figure 7D), IL-27 (Figure 7E), and TGF-β (Figure 7F) by CD1c^+^ DCs was detected by flow cytometry. Our results indicate that coculture with primary NSCLC cells downregulates the production of IL-6, IL-12, and IL-23 by CD1c^+^ DCs compared with that of CD1c^+^ DCs that are not cocultured with primary tumor cells (Figures 7A,C,D). In contrast, the secretion of IL-10 and IL-27 by CD1c^+^ DCs is enhanced after coculture with primary NSCLC cells compared with that by DCs that are not cocultured with primary NSCLC cells (Figures 7B,E). In addition, the experimental data demonstrate that the production of TGF-β by CD1c^+^ DCs incubated with primary tumor cells is similar to that by CD1c^+^ DCs that are not cocultured with primary tumor cells (Figure 7F). Since pro- and anti-inflammatory cytokines produced by DCs play an important role in regulating innate and adaptive immunity, our results suggest that primary NSCLC cells may affect DC-mediated immune function via modulating the production of pro- and anti-inflammatory cytokines *in vivo*. In addition, we also observed the expression of costimulatory molecules and production of pro-/anti-inflammatory cytokines by DCs derived from healthy donors and NSCLC patients (Supplementary Figures 2, 3). The data of absolute numbers of DC subsets mediated by CD103 and CD205 were shown in Supplementary Figure 7 (Supplementary Results).

## Discussion

We investigated the effect of NSCLC cells on development of CD1c^+^ cDCs that are reported as one of three DC populations in human peripheral blood in Ziegler-Heitbrock et al. (14). Granot et al. reported that CD1c^+^ DCs are the major typical DCs in lung-draining lymph nodes. CD1c^+^ DCs play an important role in immune surveillance in local lung tissue (19). It is unclear whether NSCLC cells affect the immune function of CD1c^+^ DCs *in vivo*. Recent research has indicated that there are multiple subsets of CD1c^+^ DCs in humans. For example, Borriello et al. found that human CD14^+^CD1c^+^ DCs were induced by lipopolysaccharide (LPS) stimulation (20). De Monte et al. found that CD1c^+^ CD207^+^ DCs were present in human tonsils (21). Data from Zaba demonstrated that CD11c^+^ CD1c^+^ DCs were found in the upper dermis and could activate T cells (22). Since CD1c^+^ DCs play an important role in innate and adaptive immunity (15, 23), it is necessary to reveal the effect of NSCLC on the differentiation of CD1c^+^ DC subsets.

Two new CD1c^+^ DC subsets (lin^−^CD1c^+^CD205^+^CD103^+^ DCs and lin^−^CD1c^+^CD205^+^CD103^−^ DCs) were identified in healthy donors and NSCLC patients (Figures 1C,D). Coculture with NSCLC cells led to the suppression of the development of the lin^−^CD1c^+^CD205^+^CD103^+^ DC subset (Figure 2C). At present, the immune function of lin^−^CD1c^+^CD205^+^CD103^+^ DCs is still unknown. CD205 is expressed on DCs and is a recognition receptor for necrotic and apoptotic cells (24). CD205^+^ DCs engulf target cells through the CD205-mediated endocytosis pathway and present antigen epitopes to CD4^+^ and CD8^+^ T cells for recognition (25–27). The number of lin^−^CD1c^+^CD205^+^ DCs in NSCLC patients was lower than that in healthy donors (Figure 1A). Our results imply that there are fewer CD205^+^ DCs in NSCLC patients. This may decrease the efficiency of the endocytosis of apoptotic and necrotic tumor cells by DCs, which may reduce antigen presentation that induces CD4^+^/CD8^+^ T cell-mediated anti-tumor immunity.

Interestingly, Yamazaki et al. reported that CD8^+^CD205^+^ splenic DCs facilitate the development of regulatory T cells (T_regs_) (28). Since there are more T_regs_ in NSCLC patients than in healthy people (29) and our data indicate that coculture with NSCLC cells elicits the development of lin^−^CD1c^+^CD205^+^ DCs (Figure 2A), NSCLC cells may facilitate the differentiation of T_regs_ via modulating the development of lin^−^CD1c^+^CD205^+^ DCs *in vivo*.

CD103^+^ DCs play an important role in the induction of anti-tumor immunity. For instance, Mittal et al. found that CD103^+^ DCs produce IL-12 via a basic leucine zipper ATF-like transcription factor 3 (BATF3)-mediated pathway to activate NK cells and inhibit tumor metastasis (30). Our results demonstrated that coculture with NSCLC cells blocks the development of lin^−^CD1c^+^CD103^+^ DCs (Figure 2B). These results suggest that NSCLC cells may inhibit NK cell-mediated anti-tumor immunity through suppressing the immune function of lin^−^CD1c^+^CD103^+^ DCs *in vivo*.

Interestingly, NSCLC cells elicit the development of lin^−^CD1c^+^CD205^+^CD103^+^ DCs derived from healthy donors (Supplementary Figure 2C) but inhibit the differentiation of lin^−^CD1c^+^CD205^+^CD103^+^ DCs derived from NSCLC patients (Figures 2C, 3C). Our results imply that DCs in NSCLC patients may be different from those isolated from healthy donors. Their biological function may be blocked due to the NSCLC-induced immune suppressive microenvironment. Future work needs to be conducted to determine the reason why NSCLC cells have different effects on the development of the CD1c^+^ DC subsets isolated from NSCLC patients and healthy donors.

DCs regulate immune function via the Signal 1, 2, and 3 transduction pathways. MHC I and II molecules on DCs bind to CTL epitopes and associate with T cell receptors (TCRs) for target cell recognition (Signal 1) (31). Furthermore, there are multiple costimulatory molecules, such as CD80 and CD86, expressed on DCs. These signal molecules bind to ligands expressed on T cells to modulate T cell activation (Signal 2) (32). For example, CD40 expressed on DCs binds to CD40L presented on T cells to initiate T cell-mediated immune responses. CD80 and CD86 expressed on DCs bind to CD28 and CD152 presented on T cells to induce T cell proliferation and are necessary for T cell survival (32, 33). In addition, DCs also produce cytokines to modulate the activation of immune cells (Signal 3) (34, 35). These are the molecular basis of the central role played by DCs in regulating the biological function of the immune system.

It is still unclear whether NSCLC cells can affect DC-mediated immune responses through regulating the protein expression of signal molecules expressed on DCs. We systemically investigated the effect of NSCLC cells on the expression of Signal 1-, 2-, and 3-associated molecules on CD1c^+^ DCs (Figures 4–7). Coculture with NSCLC cells leads to the downregulation of the expression of CD40, CD80, CD86, and HLA-DR on human CD1c^+^ DCs (Figures 4, 5). The biological features of NSCLC-incubated DCs are similar to those of tolerogenic DCs, which have been previously used for DC-mediated immunotherapy to target autoimmune diseases (36–43). Our results suggest that NSCLC cells may be able to induce tolerogenic DCs with the low expression of costimulatory molecules and MHCs so that DC-mediated immune responses that are dependent on Signal 1-, 2-, and 3-associated molecules expressed on DCs are inhibited. NSCLC-induced tolerogenic DC subsets may function as part of the cellular mechanism involved in the NSCLC-mediated immune suppressive microenvironment *in vivo*.

DCs also produce multiple cytokines to modulate immune responses (44). For example, DCs secrete several pro-inflammatory cytokines, including IL-6, IL-12, and IL-23, to facilitate T cell-mediated immune responses (45). Nizzoli et al. reported that human CD1c^+^ DCs activate cytotoxic T lymphocytes via IL-12 produced by CD1c^+^ DCs (46). Aliahmadi et al. found that human Langerhans cells with activation of the Toll-like receptor 2-mediated signal transduction pathway facilitate the development of T helper 17 (Th17) cells through the IL-1-beta, IL-23, and TGF-beta-mediated signal transduction pathways (47). Since NSCLC cells downregulate the production of IL-12 and IL-23 in CD1c^+^ DCs (Figures 6C,D), tolerogenic CD1c^+^ DCs may block T cell-mediated anti-tumor immunity via suppressing the production of IL-12 and IL-23 by CD1c^+^ DCs, which are necessary for T cell activation *in vivo*.

Both pro-inflammatory cytokines and anti-inflammatory cytokines can be produced by DCs (45). For example, DCs secrete IL-10, IL-27, and TGF-beta to modulate CD8^+^ and CD4^+^ T cell-mediated immune responses (34). Nizzoli et al. reported that CD1c^+^ DCs shape naive CD8^+^ T cell priming via IL-10-mediated signaling produced by CD1c^+^ DCs (48). Tsoumakidou et al. found that tolerogenic CD1c^+^ DCs derived from chronic obstructive pulmonary diseases (COPD) induce the generation of CD4^+^ T_regs_ through IL-10- and IL-27-induced costimulatory ligands (49). It is known that there are more T_regs_ in NSCLC patients (29). Since NSCLC cells facilitate the production of IL-10 and IL-27 in CD1c^+^ DCs (Figures 6B,E), NSCLC cells may block the activity of CD8^+^ T cells and elicit the development of CD4^+^ T_regs_ through IL-10 and IL-27 produced by CD1c^+^ DCs *in vivo*. This may be one aspect of the cellular and molecular mechanisms involved in the NSCLC-mediated immune suppressive microenvironment in NSCLC patients. We will conduct further studies of CD1c^+^ DC subset-mediated T cell responses in the future.

It is still unclear how NSCLC cells modulate the development of CD1c^+^ cDC subsets mediated by CD103 and CD205. It has been known that NSCLC cells can produce anti-inflammatory cytokines such as IL-10 and TGF-β, which may lead to tumor tolerance in NSCLC patients. In addition, NSCLC cells facilitate the production of TGF-β by DCs and elicit the development of T_reg_ in NSCLC patients so that the immune function of patients is inhibited. This probably is one of the mechanisms of immune suppressive microenvironment mediated by NSCLC *in vivo*. We will continue to investigate the molecular mechanisms of NSCLC-induced immune tolerogenic CD1c^+^ DC subsets mediated by CD103 and CD205 in the future so that the cellular mechanisms of NSCLC-mediated immune suppressive micro-environment can be further elucidated.

In summary, we investigated the effect of NSCLC on the development of CD1c^+^ DC subsets mediated by CD205 and CD103 in this project. We identified two new subpopulations of CD1c^+^ DCs: lin^−^CD1c^+^CD205^+^CD103^+^ DCs and lin^−^CD1c^+^CD205^+^CD103^−^ DCs. NSCLC cells specifically suppress the development of lin^−^CD1c^+^CD205^+^CD103^+^ DCs. In addition, NSCLC cells downregulate the expression of costimulatory molecules (CD80 and CD86) and pro-inflammatory cytokines (IL-12 and IL-23); however, NSCLC cells facilitate the secretion of anti-inflammatory cytokines (IL-10) in CD1c^+^ DCs. It can be concluded that NSCLC cells may induce the production of a tolerogenic CD1c^+^ DC subset and thereby block anti-tumor immunity *in vivo*. Tolerogenic CD1c^+^ DC subsets mediated by CD205 and CD103 may play an important role in the NSCLC-induced immune suppressive microenvironment.

## Data Availability Statement

The datasets generated for this study are available on request to the corresponding author.

## Ethics Statement

The studies involving human participants were reviewed and approved by Cas-lamvac Biotech Co., Ltd. The patients/participants provided their written informed consent to participate in this study.

## Author Contributions

YL, WX, YG, XChang, GW, and ZR conducted the experiments. LQ and XChen analyzed data and supervised project. FZ designed the experiments, supervised the research, and wrote the manuscript.

### Conflict of Interest

YL, WX, YG, XChang, GW, ZR, LQ, XChen, and FZ were employed by the CAS Lamvac Biotech Co., Ltd.

## Abbreviations

APC: Allophycocyanin
CD: Cluster of differentiation
COPD: Chronic obstructive pulmonary diseases
DC: Dendritic cell
FCS: Fetal Calf Serum
Fig: Figure
GM–CSF: Granulocyte-macrophage colony-stimulating factor
IL: Interleukin
Lin: Lineage
LPS: Lipopolysaccharide
2-ME: 2-mercaptoethanol
NSCLC: Non-small cell lung cancer
PBS: Phosphate-buffered saline
PBMCs: Peripheral blood mononuclear cells
SD: Standard deviation
SEM: Standard error of arithmetic mean
Th: Helper T cells
T_regs_: Regulatory T cells.
