# Supplementary material for: Non-small Cell Lung Cancer Cells Modulate the Development of Human CD1c+ Conventional Dendritic Cell Subsets Mediated by CD103 and CD205
Source: Front Immunol. 2019 Dec 10;10:2829. doi: 10.3389/fimmu.2019.02829 (PMC6914740; doi:10.3389/fimmu.2019.02829)
Supplement: Supplementary file 1 [file Data_Sheet_1.doc]

**Supplementary Results:**

1. The expression of CD40 and CD86 on CD1c+ DCs is downregulated in NSCLC patients.

Since DC-mediated immune responses are dependent on signal molecules such as CD40, CD80, CD86 and HLA-DR that are expressed on DCs, NSCLC cells may affect the immune function of DCs by modulating the expression of signal molecules on DCs. To test whether NSCLC cells regulate the expression of CD40, CD80, CD86 and HLA-DR on DCs, the protein expression of signal molecules, including CD40, CD80, CD86 and HLA-DR, on CD1c+ DCs derived from the peripheral blood of NSCLC patients and healthy donors was detected by flow cytometry. The experimental data show that the expression of CD40 and CD86 on CD1c+ DCs derived from NSCLC patients is lower than that on CD1c+ DCs isolated from healthy donors (Supplementary Figs. 2A and 2C). In contrast, there is no significant difference between NSCLC patients and healthy donors in terms of the expression of CD80 and HLA-DR on CD1c+ DCs (Supplementary Figs. 2B and 2D). Our results suggest that NSCLC cells may affect CD1c+ DC-mediated immune responses by downregulating the expression of CD40 and CD86 on CD1c+ DCs.

2. The production of IL-10 and IL-27 in CD1c+ DCs is enhanced in NSCLC patients.

Since DCs also modulate immune responses through the secretion of pro- and anti-inflammatory cytokines, NSCLC cells may affect DC-mediated immune responses via regulating the production of cytokines by DCs. To detect whether NSCLC cells affect the production of pro- and anti-inflammatory cytokines by CD1c+ DCs, PBMCs were isolated from NSCLC patients and healthy donors. Lin-CD1c+ cells were gated, and the production of IL-6, IL-10, IL-12, IL-23, IL-27 and TGF-β were detected by flow cytometry. The experimental data indicate that there is no significant difference between NSCLC patients and healthy donors in the production of IL-6, IL-12, IL-23 and TGF-β (Supplementary Figs. 3A, 3C, 3D and 3F). In contrast, the production of IL-10 and IL-27 in CD1c+ DCs derived from NSCLC patients is higher than that in CD1c+ DCs isolated from healthy donors (Supplementary Figs. 3B and 3E). Our results imply that NSCLC cells may affect DC-mediated immune responses through eliciting the production of IL-10 and IL-27.

3. Coculture with NSCLC cell line H-1299 leads to the downregulation of the protein expression of signal molecules on CD1c+ DCs isolated from healthy donors.

Since our data show that the expression and secretion of some signal molecules and cytokines in CD1c+ DCs derived from NSCLC patients are abnormal compared with those in CD1c+ DCs isolated from healthy donors (Supplementary Figs. 2-3), we proposed that NSCLC cells may affect the expression of signal molecules and cytokines in DCs. To test this hypothesis, DCs isolated from three healthy donors were incubated with NSCLC H-1299 cells or were not co-cultured with H-1299 cells as a control. The protein expression of signal molecules, including CD40 (Supplementary Fig. 4A), CD80 (Supplementary Fig. 4B), CD86 (Supplementary Fig. 4C) and HLA-DR (Supplementary Fig. 4D), were detected by flow cytometry. Our results indicate that the expression of CD40, CD80, CD86 and HLA-DR on CD1c+ DCs derived from healthy donors is downregulated after co-culture with the H-1299 cell line compared with that expressed on CD1c+ DCs without incubation with H-1299 cells (Supplementary Fig. 4). This implies that H-1299 tumor cells may block the immune function of CD1c+ DCs through inhibiting the expression of signal molecules such as CD40, CD80, CD86 and HLA-DR on DCs.

5. H-1299 tumor cells modulate the production of pro- and anti-inflammatory cytokines by CD1c+ DCs derived from healthy donors.

DCs regulate immune function through the secretion of pro- and anti-inflammatory cytokines such as IL-6, IL-12, IL-10 and IL-27. Our data demonstrated that the production of IL-10 and IL-27 by CD1c+ DCs derived from NSCLC patients is greater than that by CD1c+ DCs isolated from healthy donors (Supplementary Fig. 2). We hypothesized that NSCLC cells may affect the immune function of DCs via regulating the production of pro- or anti-inflammatory cytokines by DCs. To test this hypothesis, DCs derived from healthy donors were cocultured with H-1299 tumor cells or were not cocultured with H-1299 cells as a control. The production of IL-6 (Supplementary Fig. 5A), IL-10 (Supplementary Fig. 5B), IL-12 (Supplementary Fig. 5C), IL-23 (p19) (Supplementary Fig. 5D), IL-27 (Supplementary Fig. 5E) and TGF-β (Supplementary Fig. 5F) by CD1c+ DCs was detected by flow cytometry. Our data show that the production of IL-6, IL-10 and IL-27 by CD1c+ DCs is increased after incubation with H-1299 cells (Supplementary Figs. 5A, 5B and 5E) compared with that by CD1c+ DCs without coculture with H-1299 tumor cells. In contrast, the secretion of IL-12 and IL-23 by CD1c+ DCs is downregulated after coculture with H-1299 cells (Supplementary Figs. 5C and 5D) compared with that by CD1c+ DCs without incubation with H-1299 cells. In addition, there is no significant difference in the production of TGF-β in CD1c+ DCs incubated with H-1299 cells and CD1c+ DCs that are not co-cultured with H-1299 tumor cells (Supplementary Fig. 5F). Our results suggest that H-1299 tumor cells may affect the immune function of CD1c+ DCs through modulating the production of pro- and anti-inflammatory cytokines, such as IL-6, IL-10, IL-12, IL-23 and IL-27.

6. H-1299 tumor cells elicit the development of CD1c+ DC subsets isolated from healthy donors mediated by CD205 and CD103.

Multiple DC subpopulations have been identified according to their different biological functions, such as inflammatory and tolerogenic DCs. Our data indicated that there are two novel DC subsets, CD1c+CD205+CD103+ DCs and CD1c+CD205+CD103- DCs, in healthy donors and NSCLC patients (Fig. 1). The development of the CD1c+CD205+CD103+ DC subset was downregulated in NSCLC patients compared with that in healthy donors (Fig. 1C). We proposed that NSCLC cells may specifically regulate the development of some DC subsets. To test this hypothesis, DCs derived from healthy donors were incubated with H-1299 tumor cells. The frequency of the CD1c+ DC subset mediated by CD205 (Supplementary Fig. 6A) and CD103 (Supplementary Fig. 6B) was detected by flow cytometry. Our results show that coculture with H-1299 tumor cells upregulates CD205 and CD103 expression on CD1c+ DCs (Supplementary Figs. 6A and 6B). The development of the CD1c+CD205+CD103+ DC and CD1c+CD205+CD103- DC subsets is facilitated after incubation with H-1299 cells compared with that of CD1c+CD205+CD103+ and CD1c+CD205+CD103- DCs that are not cocultured with H-1299 cells (Supplementary Figs. 6C and 6D). Our data imply that H-1299 cells may specifically modulate the development of the DC subsets derived from healthy donors mediated by CD205 and CD103.

**Supplementary Figure Legends:**

**Supplementary Figure 1:** The strategy of CD1c^+^ DC population gating. Cells were isolated from one NSCLC patient and stained by human linage and CD1c antibodies. Lin-CD1c^+^ cells were gated. Isotype control (Cells were derived from one healthy donor and incubated with isotype control antibodies.) was also shown.

**Supplementary Figure 2:** The protein expression of CD40, CD80, CD86 and HLA-DR on CD1c^+^ DCs derived from NSCLC patients and healthy donors. PBMCs were isolated from NSCLC patients and healthy donors. Cells were stained with human CD1c, CD40, CD80, CD86, HLA-DR and lineage antibodies. A flow cytometry assay was conducted, and Lin^-^CD1c^+^ cells were gated. The protein expression of CD40 (A), CD80 (B), CD86 (C) and HLA-DR (D) on CD1c+ DCs is shown. The error bars indicated in this figure represent the mean and SD of quadruplicate determinations of the mean fluorescence identities in one experiment (*P<0.05, n=4, *t* test).

**Supplementary Figure 3:** The production of pro- and anti-inflammatory cytokines by CD1c^+^ DCs isolated from NSCLC patients and healthy donors. PBMCs were derived from 4 NSCLC patients and 4 healthy donors. Cells were stained with human CD1c, lineage, IL-6, IL-10, IL-12, IL-23 (p19), IL-27 and TGF-β antibodies. A flow cytometry assay was carried out, and lin-CD1c^+^ cells were gated. Cytokine production, including IL-6 (A), IL-10 (B), IL-12 (C), IL-23 (p19) (D), IL-27 (E) and TGF-β (F), by CD1c^+^ DCs was determined. The error bars shown in this figure show the mean and SD of quadruplicate determinations of frequency from one experiment (*P<0.05, n=4, *t* test).

**Supplementary Figure 4:** H-1299 tumor cells suppress the protein expression of signal molecules on CD1c^+^ DCs derived from healthy donors. PBMCs were isolated from 3 healthy donors and induced the development of DCs *in vitro*. DCs were incubated with H-1299 tumor cells for 24 hrs or were not co-cultured wit H-1299 cells as a control. DCs were stained with human CD1c, CD40, CD80, CD86, HLA-DR and linage antibodies. A flow cytometry assay was conducted, and Lin^-^CD1c^+^ cells were gated. Protein expression of CD40 (A), CD80 (B), CD86 (C) and HLA-DR (D) on CD1c^+^ DCs is shown. The error bars indicated in this figure represent the mean and SD of triplicate determinations of the mean fluorescence identities from one experiment (*P<0.05, n=3, *t* test).

**Supplementary Figure 5:** H-1299 tumor cells modulate the production of pro- and anti-inflammatory cytokines by CD1c^+^ DCs derived from healthy donors. PBMCs were derived from 3 healthy donors. PBMCs were cultured in DC medium for 8 days to induce the development of DCs. DCs were stained with human CD1c, linage, IL-6, IL-10, IL-12, IL-23 (p19), IL-27 and TGF-β antibodies. A flow cytometry assay was carried out, and lin^-^CD1c^+^ cells were gated. Cytokine production, including IL-6 (A), IL-10 (B), IL-12 (C), IL-23 (p19) (D), IL-27 (E) and TGF-β (F), by CD1c+ DCs is shown. The error bars shown in this figure represent the mean and SD of triplicate determinations of cytokine production by CD1c^+^ DCs in one experiment (* P<0.05, n = 3, *t* test).

**Supplementary Figure 6**: H-1299 tumor cells facilitate the development of CD1c^+^ DC subsets derived from healthy donors mediated by CD205 and CD103. PBMCs from 3 healthy donors were collected and stained with human CD1c, CD103, CD205 and linage antibodies. Lin^-^CD1c^+^ cells were gated. The protein expression of CD205 (A) and CD103 (B) on CD1c^+^ DCs and the frequency of the CD1c^+^CD205^+^CD103^+^ DC subset (C) and the CD1c^+^CD205^+^CD103^-^ DC subpopulation (D) were determined. The error bars shown in this figure represent the mean and SD of triplicate determinations of the frequency of the CD1c^+^ subpopulations from one experiment (*P<0.05, n=3, *t* test).

**Supplementary Figure 7:** Statistical analysis of absolute cell number of CD1c^+^ DC subsets mediated by CD205 and CD103. (A-D): Cell numbers of CD1c^+^ DC subpopulations isolated from healthy donors and NSCLC patients (n=4). (E-H): Cell numbers of CD1c^+^ DC subsets derived from healthy donors cocultured with H-1299 or without incubation with tumor cell line (n=3). (I-L): Cell numbers of CD1c^+^ DC subpopulations incubated with H-1299 or without coculture with tumor cell line derived from NSCLC patients (n=3). (M-P): Cell numbers of CD1c^+^ DC subsets cocultured with primary NSCLC cells or without incubation with primary NSCLC cells (n=2). The error bars indicated in this figure represent mean and SD of determinations of CD1c^+^ DC subset cell numbers (*P<0.05, *t* test).


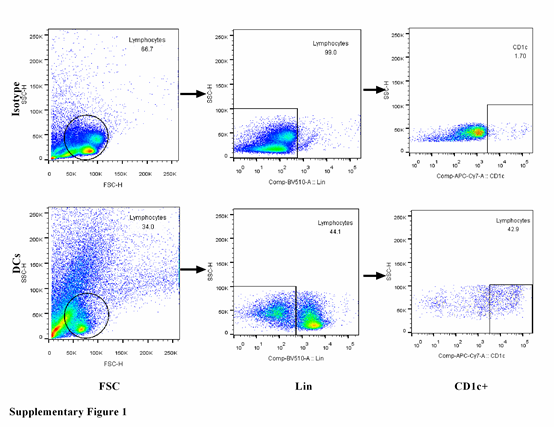


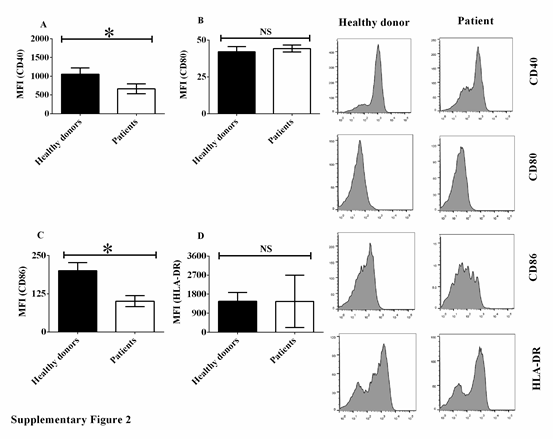


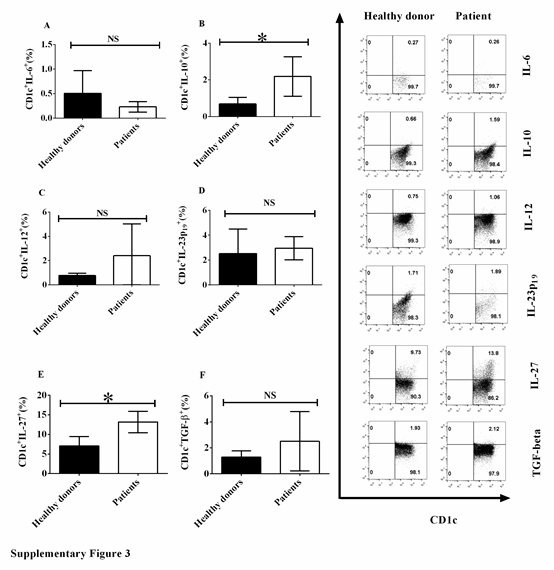


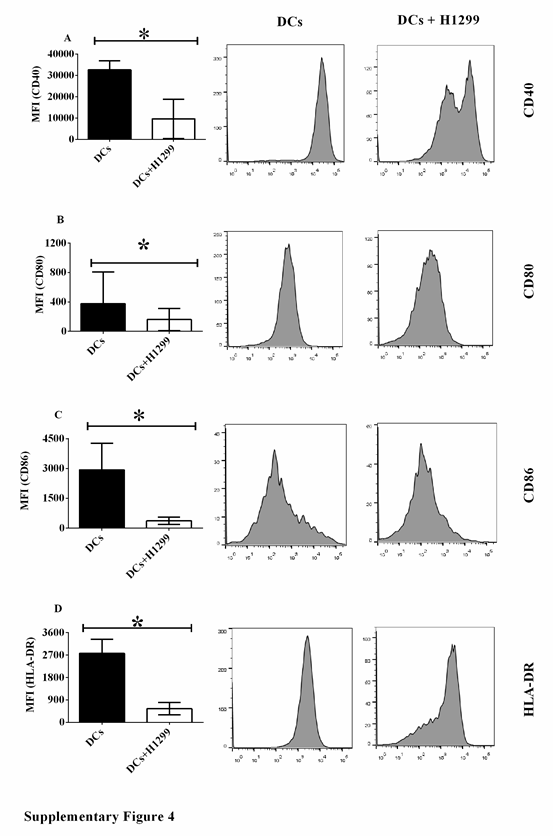


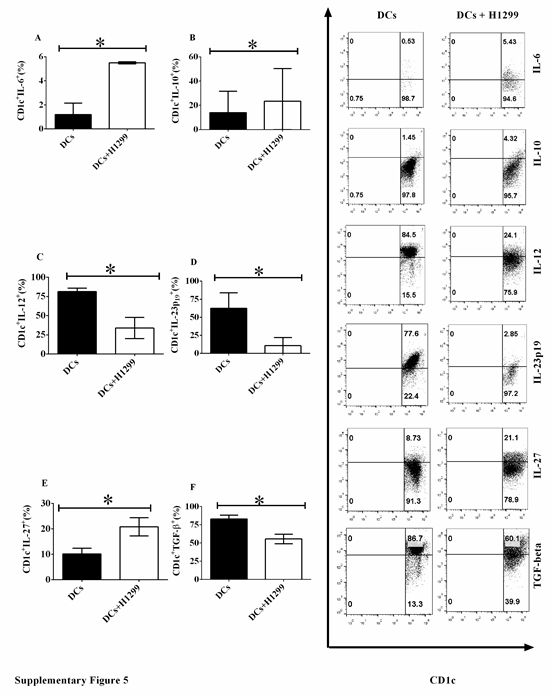


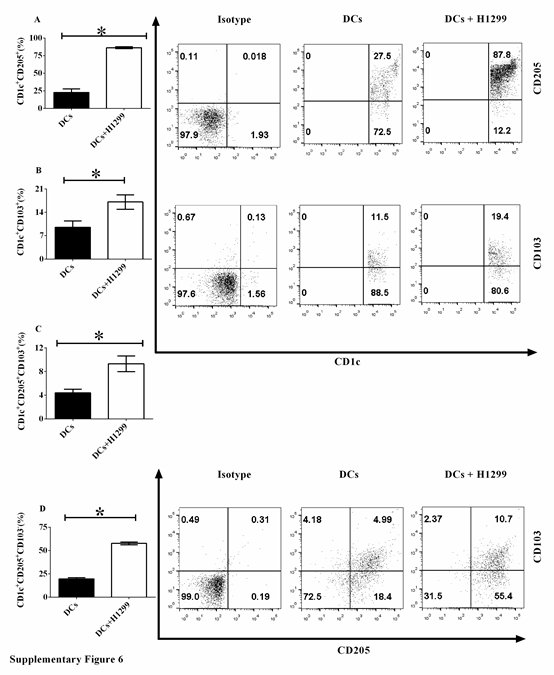


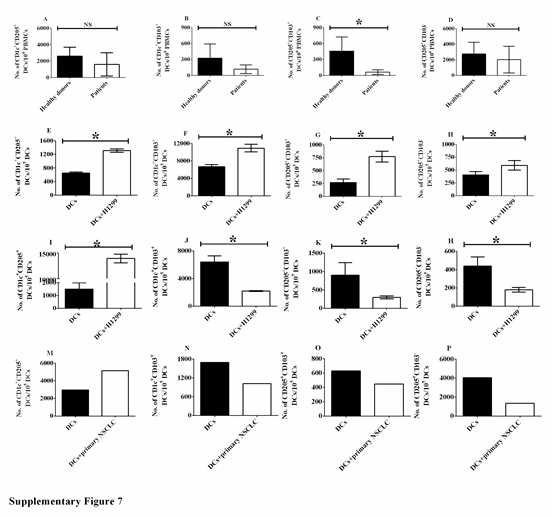


| NSCLC patients (n=7) | Healthy donors (n=7) |
| --- | --- |

| NSCLC patients (n=7) | Healthy donors (n=7) |
| --- | --- |
| Ethnicity Chinese  Gender 4 males  3 females  Age  (Mean+/-SD) 53.29 +/- 10.71  Concomitant  Disease None  Histology 7 adenocarcinoma  Stage 1 IIIA; 6 IVA  Chemotherapy 2 chemo-therapy  5 none  Radiotherapy 1 radiotherapy  6 none  Targeted  therapies 6 targeted therapies  1 none  Immunotherapy None | Chinese  5 males  2 females  32.14 +/- 4.86  None  None  None  None  None  None  None |

Table-1: Characteristics of patients with non-small-cell lung cancer and healthy donors
